# Maternal, social and abiotic environmental effects on growth vary across life stages in a cooperative mammal

**DOI:** 10.1111/1365-2656.12149

**Published:** 2013-11-18

**Authors:** Sinead English, Andrew W Bateman, Rafael Mares, Arpat Ozgul, Tim H Clutton-Brock

**Affiliations:** 1 Large Animal Research Group, Department of Zoology, University of CambridgeCambridge, CB2 3EJ; 2 Mammal Research Institute, Department of Zoology and Entomology, University of PretoriaPretoria, 0002, South Africa

**Keywords:** body mass, carry-over effects, cooperative breeding, growth, life history

## Abstract

1. Resource availability plays a key role in driving variation in somatic growth and body condition, and the factors determining access to resources vary considerably across life stages. Parents and carers may exert important influences in early life, when individuals are nutritionally dependent, with abiotic environmental effects having stronger influences later in development as individuals forage independently.
2. Most studies have measured specific factors influencing growth across development or have compared relative influences of different factors within specific life stages. Such studies may not capture whether early-life factors continue to have delayed effects at later stages, or whether social factors change when individuals become nutritionally independent and adults become competitors for, rather than providers of, food.
3. Here, we examined variation in the influence of the abiotic, social and maternal environment on growth across life stages in a wild population of cooperatively breeding meerkats. Cooperatively breeding vertebrates are ideal for investigating environmental influences on growth. In addition to experiencing highly variable abiotic conditions, cooperative breeders are typified by heterogeneity both among breeders, with mothers varying in age and social status, and in the number of carers present.
4. Recent rainfall had a consistently marked effect on growth across life stages, yet other seasonal terms only influenced growth during stages when individuals were growing fastest. Group size and maternal dominance status had positive effects on growth during the period of nutritional dependence on carers, but did not influence mass at emergence (at 1 month) or growth at independent stages (>4 months). Pups born to older mothers were lighter at 1 month of age and subsequently grew faster as subadults. Males grew faster than females during the juvenile and subadult stage only.
5. Our findings demonstrate the complex ways in which the external environment influences development in a cooperative mammal. Individuals are most sensitive to social and maternal factors during the period of nutritional dependence on carers, whereas direct environmental effects are relatively more important later in development. Understanding the way in which environmental sensitivity varies across life stages is likely to be an important consideration in predicting trait responses to environmental change.

## Introduction

Growth is an important life-history process, influencing a range of later fitness-related traits such as age and size at maturity, total reproductive output, and the onset and rate of senescence (Millar & Zammuto 1983; Stearns 1992; Charnov 2004; Monaghan *et al.* 2008). Intraspecific variation in growth is therefore a primary determinant of the material on which natural selection acts. While some of this variation may be due to genetic differences (Dmitriew 2011), growth is a highly plastic trait that is sensitive to the availability of resources in the environment (Nylin & Gotthard 1998). Food availability may be influenced both by abiotic environmental effects, such as rainfall or seasonal fluctuation in resources, and by social factors whereby individuals cooperate or compete with conspecifics resulting in increased or decreased access to food, respectively.

Most studies have either considered how one specific factor influences growth across development (e.g., LeBlanc, Festa-Bianchet & Jorgenson 2001), or have compared different factors within specific developmental stages (e.g., Ridley 2007). Sensitivity to environmental factors is likely to vary across life stages, however. In the early development of altricial species, growth may be strongly influenced by the behaviour and condition of carers, who often buffer young against direct environmental effects (Cadby, Jones & Wapstra 2011). Later in life, when individuals are foraging independently and competing with conspecifics over food, growth may be more directly influenced by abiotic and density-dependent environmental factors affecting resource availability (LeBlanc, Festa-Bianchet & Jorgenson 2001). Nevertheless, early environment effects may continue to exert delayed phenotypic consequences at later-life stages (e.g., Auer *et al.* 2012). One reason for such delayed effects is that individuals whose growth is stunted at one period of development may attempt to compensate by increasing their growth rates later on (Hector & Nakagawa 2012). A systematic analysis of the relative influence of several factors on growth across different stages will provide important insights into the mechanisms underlying population responses to environmental change.

Cooperatively breeding vertebrates, where non-breeding individuals help raise the young of others, offer a unique opportunity to investigate changes in factors affecting growth across life stages. Abiotic environmental effects on growth are likely to be striking as they are typically found in harsh and unpredictable environments (Jetz & Rubenstein 2011). Cooperative breeders also have a protracted stage of juvenile dependence on adults (Langen 2000). During this stage, carers are likely to have important influences on growth of young (Russell *et al.* 2002; Ridley 2007), potentially shielding them from harsh effects of the abiotic environment (Covas, du Plessis & Doutrelant 2008). There is high heterogeneity in both the maternal and social environment (Russell & Lummaa 2009), however, which may determine the extent of this buffering effect. For example, pups born to older females of reduced quality (Hart & Monnin 2006; Sharp & Clutton-Brock 2010) or stressed subordinate mothers (Dloniak, French & Holekamp 2006; Young *et al.* 2006) may experience poor growth conditions. Beyond nutritional independence, the effect of helpers should decline as abiotic environmental effects become more relevant to individual foraging success. Indeed, rather than increasing growth through providing food to individuals, other helpers may reduce growth as a result of food competition. To our knowledge, there has been no direct comparison of the relative influence of abiotic, social and maternal environmental effects on development from birth until adulthood in a cooperative vertebrate.

Here, we investigate the changing influence of environmental conditions on growth in a wild population of cooperative meerkats (*Suricata suricatta*). Meerkats live in arid regions of southern Africa characterized by stochastic rain patterns (Doolan & Macdonald 1996). Group sizes vary between 3 and 50 individuals (Clutton-Brock, Hodge & Flower 2008), with a dominant pair monopolizing most within-group reproduction (Griffin *et al.* 2003). Previous work on growth has found that maternal factors influence early condition before nutritional independence (Russell *et al.* 2002) and that group size and rainfall affect subsequent growth (Clutton-Brock *et al.* 2001; Russell *et al.* 2002). A recent model of lifetime growth in this species demonstrated the importance of rain and season across development and found different patterns of growth before and after independence (English, Bateman & Clutton-Brock 2012). Beyond considering direct environmental effects, this study did not explore the specific mechanisms driving individual variation in growth.

Our aim in this study was to examine in detail the relative influence of a suite of abiotic, social and maternal environmental factors on mass and growth at several distinct stages of development between birth and adulthood. Specifically, we were interested in whether abiotic environmental effects were weaker in early life when pups are dependent on mothers and helpers for food. We also wanted to test whether maternal factors exerted delayed effects on their development beyond the stage of nutritional dependence. At later stages of nutritional independence, we also expected a switch in the effect of social factors, when helpers may be perceived more as competitors than cooperators.

## Materials and methods

### Study site and species

This study was conducted using long-term data from a wild population of meerkats inhabiting private ranch land in the South African Kalahari Desert (26°58′S, 21°49′E). All individuals in the population were tagged with unique subcutaneous transponder chips and were identifiable in the field through dye marks on their fur. Groups were visited approximately three times per week and all life-history events, including births, deaths, immigration and emigration, were recorded. Further details on the study site and population are described elsewhere (Clutton-Brock *et al.* 1998; Russell *et al.* 2002). In this study, factors affecting body mass and growth were investigated between birth and 18 months of age in a total of 1378 individuals from 119 mothers in 26 social groups, born between January 1998 and December 2009.

### Factors affecting growth across development

Five separate analyses were conducted to investigate factors influencing mass (first stage) or growth (all other stages) in the following life stages: (i) ‘emergence’, at 1 month of age (when pups are first weighed, shortly after emerging from the natal burrow); (ii) ‘pups’, between 1 and 3 months of age (when individuals are still nutritionally dependent on adults); (iii) ‘juveniles’, between 4 and 6 months of age (when individuals are foraging independently, yet contribute little to cooperative care); (iv) ‘subadults’ between 10 and 12 months of age (when individuals are sexually mature and have started helping); and (v) ‘adults’, between 16 and 18 months of age (beyond which age few individuals remain in their natal group as subordinates). Two-month, fixed windows for growth were selected to assess the effects of short-term fluctuations in abiotic and social environmental factors and to compare them across the different stages of development. While meerkat growth is nonlinear overall, best described by a modified monomolecular curve (English, Bateman & Clutton-Brock 2012), linear approximations of growth on two-month time windows allowed for straightforward assessment of relevant effects (see Fig. S1, Supporting information).

#### Body mass and growth measurements

Mass measurements were obtained without the need for capture, as most individuals (>95%) in the population were trained to step onto a top-pan electronic scale in return for a small reward (<1 g) of egg or water. In this study, pre-foraging mass measurements taken in the morning were used, to avoid any short-term fluctuations in mass due to variable foraging success. To avoid error due to missing data or variation in sampling effort, an interpolated monthly mass measure was calculated for individuals for each age in months (for a similar approach, see Ozgul *et al.* 2010). This monthly measure was calculated by first conducting linear mixed-effect models for all individuals including mass measurements for 1 month before and after each monthly age, with age and age^2^ as fixed-effect terms, and individual as a random term. A quadratic term of age was included to account for potential deceleration of growth across the period. These models were then used to estimate a best linear unbiased predictor for each individual's mass for its exact monthly age, conditional both on the fixed-effect terms and individual-level variation. Growth measures were calculated as the difference between monthly mass measures at the appropriate ages. All analyses on growth accounted for mass at the start of the period of interest.

#### Abiotic factors

Previous work on meerkats has demonstrated that long-term growth is influenced by both season and rain (English, Bateman & Clutton-Brock 2012). A sine-plus-cosine function was included to account for intra-annual seasonal periodicity, by fitting two coefficients multiplied by sin(2·π·day/365·25) and cos(2·π·day/365·25), respectively, where ‘day’ represents the day-of-year when an individual turned the end-age of the life stage in question. Total rainfall in the two-month window prior to the mid-point of the focal period was also included. Rainfall data were obtained from the NASA GES DISC (Goddard Earth Sciences Data and Information Services Center) Giovanni online data system (described in Acker & Leptoukh 2007).

#### Social factors

The effects of both nutritionally dependent and independent group members on growth were considered by including the number of individuals younger than 3 months of age (number of pups) and the number of individuals over 6 months of age (number of adults, i.e. potential helpers), as well as a quadratic term on the latter to account for potential negative effects of resource competition in large groups. Mean values during the two-month window prior to the mid-point of the focal period were used in all analyses.

#### Maternal and individual factors

Maternal age (in days) and dominance status at birth were both included in all analyses. A quadratic term of maternal age was also considered, to test for effects of senescence (Sharp & Clutton-Brock 2010). Maternal dominance status was assessed primarily through field observation, as one female (usually the dominant) tended to give birth at a time. In the rare cases where several females bred at the same time, maternity was inferred based on genetic data (details on molecular genetic analysis are described in Nielsen *et al.* 2012). The focal individual's sex was also included in order to assess whether sex differences, if any, emerge across development in this relatively size-monomorphic species.

### Statistical analysis

Linear mixed models, created in MCMCglmm (v. 2.16, Hadfield 2010) in R (v. 2.15, R Core Team 2012), were used to analyse the data. Continuous predictor variables were mean-centred and standardized for each data set for a particular growth period, for ease of comparison within and among models. All predictor variables were retained in each model, as our aim was not to derive the best predictive model of growth at each stage, but to compare the relative influence of predictor variables across different stages. MCMCglmm was therefore used calculate 95% credible intervals for each fixed parameter. MCMCglmm iterations were run with default inverse Wishart priors set at V = 1 and nu = 0·002 for all random effects (Gelman & Hill 2007). For each model, three separate chains were run and convergence of model parameters assessed by calculating the Gelman–Rubin statistic (Gelman 1996). For each chain, 2 000 000 iterations were run, with samples taken every 500 iterations and the first 1 500 000 removed as burn-in. This resulted in 1000 samples, which were used to calculate posterior modes and 95% credible intervals for the parameters. When credible intervals did not span zero, the parameter's effect was deemed to be statistically significant. Collinearity among predictor variables was assessed prior to analysis by calculating variance inflation factors (Zuur *et al.* 2009). As these were all less than 1·8, collinearity was deemed unlikely to affect the results. Random intercept terms for litter identity, mother identity and group identity were included in all models. The former two terms accounted for unexplained variation based on common genetic and environmental factors shared by littermates and individuals born to the same mother. Group identity accounted for unexplained variation affecting members of the same group. Repeatability estimates and 95% credibility intervals for each random-effect term were calculated following Nakagawa & Schielzeth (2010).

## Results

### Abiotic factors

**Fig 1:**
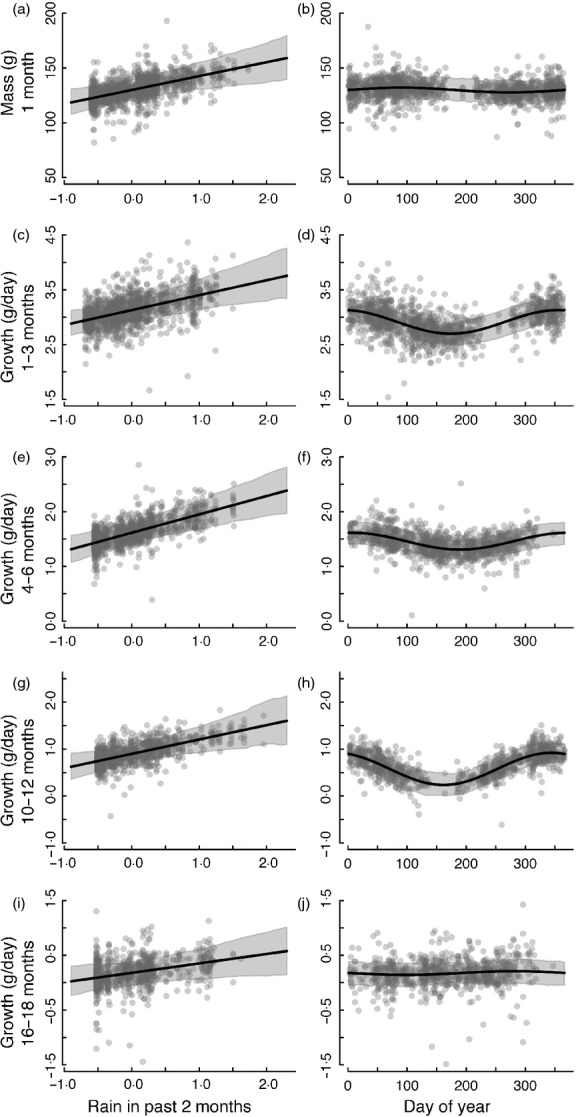
Abiotic factors affecting (a, b) mass at 1 month and (c–j) growth at subsequent stages. Left panel displays effect of rain (standardized) on mass at 1 month, and right panel displays the effect of season. Shown are the predicted mean effects and 95% credible intervals for the model fit to each period. The grey points are partial residuals accounting for other terms in the model.

Rainfall in the past 2 months had a statistically significant, positive effect on mass and growth at all stages (Tables1 and 2, Fig. 1). There were marked seasonal effects on growth at the pup, juvenile and subadult stage, but not on mass at emergence or growth as adults (Tables1 and 2, Fig. 1). Growth peaked during the hot-wet months of the year (October–March) and was lowest during the cold-dry season (April–September).

**Table 1:** Posterior means and lower and upper 95% higher posterior density credibility intervals (LCI, UCI) for all predictors, random-effect variance parameters and a breakdown of sample size at each level of random effect for the model investigating variation in body mass at 1 month of age. The probability that a fixed-effect estimate does not differ from zero is provided by the pMCMC values. Fixed effects (apart from the intercept) with pMCMC < 0.05 are highlighted in bold. For categorical variables, the level estimated, relative to the baseline intercept level, is provided in parentheses (‘D’ denotes dominant and ‘F’ denotes female)

Predictors	Posterior mean [LCI, UCI]	pMCMC
Intercept	129·88 [120·751, 138·681]	0·001
Season (sine)	2·169 [−3·427, 7·231]	0·436
Season (cosine)	0·031 [−3·565, 4·149]	0·992
Rain in past 60 days	**12·676 [5·223, 20·748]**	**0·001**
Number of adults	−6·024 [−13·064, 0·843]	0·09
(Number of adults)^2^	−6·923 [−14·693, 0·468]	0·082
Number of pups	−4·087 [−9·781, 1·393]	0·164
Maternal status (D)	−2·687 [−10·65, 6·427]	0·536
Maternal age	−5·783 [−13·891, 2·512]	0·158
(Maternal age)^2^	**−8·915 [−15·621, −1·437]**	**0·018**
Sex (F)	0·083 [−1·087, 1·331]	0·92

Variance	Posterior mean [LCI, UCI]	N
Litter	447·816 [376·682, 525·846]	379
Mother	196·968 [99·663, 314·736]	119
Group	77·099 [0·000, 203·632]	26
Residual	101·937 [93·365, 111·072]	1378

**Table 2:** Posterior means and lower and upper 95% higher posterior density credibility intervals (LCI, UCI) for all predictors, random-effect variance parameters and sample sizes at each level of random effect for the four models investigating factors affecting growth between 1 and 18 months of age. The probability that a fixed-effect estimate does not differ from zero is provided by the pMCMC values. Fixed effects (apart from the intercept) with pMCMC < 0.05 are highlighted in bold. For categorical variables, the level estimated, relative to the baseline intercept level, is provided in parentheses (‘D’ denotes dominant and ‘F’ denotes female)

Predictors	Growth 1–3 months		Growth 4–6 months		Growth 10–12 months		Growth 16–18 months	
	Posterior mean [LCI, UCI]	pMCMC	Posterior mean [LCI, UCI]	pMCMC	Posterior mean [LCI, UCI]	pMCMC	Posterior mean [LCI, UCI]	pMCMC
Intercept	2·915 [2·728, 3·109]	0·001	1·461 [1·312, 1·625]	0·001	0·579 [0·398, 0·782]	0·001	0·176 [0·008, 0·361]	0·048
Mass at start	**0·224 [0·142, 0·301]**	**0·001**	**0·176 [0·119, 0·236]**	**0·001**	**−0·064 [−0·122, −0·005]**	**0·028**	**−0·135 [−0·213, −0·057]**	**0·001**
Season (sine)	−0·031 [−0·144, 0·094]	0·632	0·017 [−0·095, 0·142]	0·754	−0·121 [−0·265, 0·023]	0·112	−0·033 [−0·161, 0·091]	0·638
Season (cosine)	**0·214 [0·14, 0·302]**	**0·001**	**0·153 [0·068, 0·242]**	**0·002**	**0·320 [0·207, 0·421]**	**0·001**	0·004 [−0·086, 0·091]	0·932
Rain in past 60 days	**0·273 [0·123, 0·433]**	**0·001**	**0·335 [0·167, 0·499]**	**0·001**	**0·306 [0·104, 0·525]**	**0·001**	**0·172 [0·006, 0·342]**	**0·052**
Number of adults	**0·151 [0·012, 0·280]**	**0·02**	−0·101 [−0·231, 0·032]	0·126	0·091 [−0·047, 0·234]	0·19	−0·015 [−0·133, 0·089]	0·78
(Number of adults)^2^	0·092 [−0·085, 0·246]	0·294	−0·007 [−0·193, 0·153]	0·942	−0·066 [−0·281, 0·135]	0·518	−0·065 [−0·274, 0·146]	0·532
Number of pups	−0·112 [−0·238, 0·008]	0·088	0·036 [−0·092, 0·165]	0·592	−0·078 [−0·210, 0·062]	0·3	0·084 [−0·057, 0·218]	0·24
Maternal status (D)	**0·204 [0·020, 0·377]**	**0·022**	−0·001 [−0·138, 0·147]	0·998	−0·067 [−0·230, 0·11]	0·466	0·062 [−0·095, 0·236]	0·458
Maternal age	0·035 [−0·153, 0·19]	0·678	−0·025 [−0·179, 0·119]	0·740	−0·07 [−0·258, 0·105]	0·456	0·156 [−0·011, 0·334]	0·088
(Maternal age)^2^	−0·051 [−0·212, 0·116]	0·542	0·062 [−0·090, 0·209]	0·420	**0·175 [−0·008, 0·33]**	**0·044**	−0·047 [−0·191, 0·118]	0·572
sex (F)	−0·018 [−0·053, 0·017]	0·332	**−0·055 [−0·087, −0·025]**	**0·002**	**−0·054 [−0·089, −0·023]**	**0·004**	−0·015 [−0·066, 0·037]	0·552

Variance	Posterior mean [LCI, UCI]	N	Posterior mean [LCI, UCI]	N	Posterior mean [LCI, UCI]	N	Posterior mean [LCI, UCI]	N
Litter	0·243 [0·200, 0·289]	365	0·231 [0·192, 0·27]	348	0·302 [0·255, 0·358]	322	0·192 [0·15, 0·227]	292
Mother	0·024 [0·001, 0·053]	113	0·004 [0·000, 0·013]	110	0·004 [0·000, 0·013]	102	0·004 [0·000, 0·011]	96
Group	0·015 [0·000, 0·042]	25	0·003 [0·000, 0·009]	24	0·019 [0·001, 0·052]	26	0·004 [0·000, 0·011]	22
Residual	0·078 [0·071, 0·087]	1252	0·053 [0·048, 0·058]	1115	0·051 [0·046, 0·057]	963	0·093 [0·081, 0·105]	776

### Social factors

**Fig 2:**
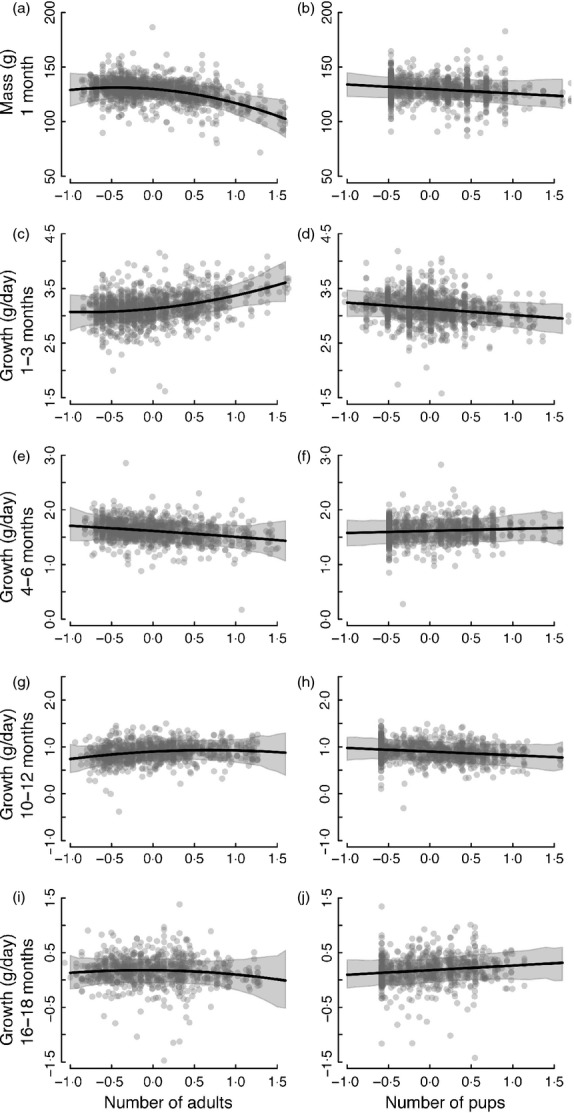
Social factors affecting (a, b) mass at 1 month and (c–j) growth at subsequent stages, with left panel displaying the effect of number of adults (standardized) on mass at 1 month and right panel displaying the effect of pups (standardized). Shown are the predicted mean effects and 95% credible intervals for the model fit to each period. The grey points are partial residuals accounting for other terms in the model.

The number of adults in a group had a positive effect on pup growth rates (Table 2, Fig. 2), but did not influence growth in any other stages. There was, however, a trend for a quadratic effect on mass at emergence (Fig. 2), with pups born lighter in very large groups. Individuals born in larger litters suffered reduced growth as pups (Table 2, Fig. 2), but the number of pups in the group did not otherwise influence mass at emergence or growth in later stages.

### Maternal factors

**Fig 3:**
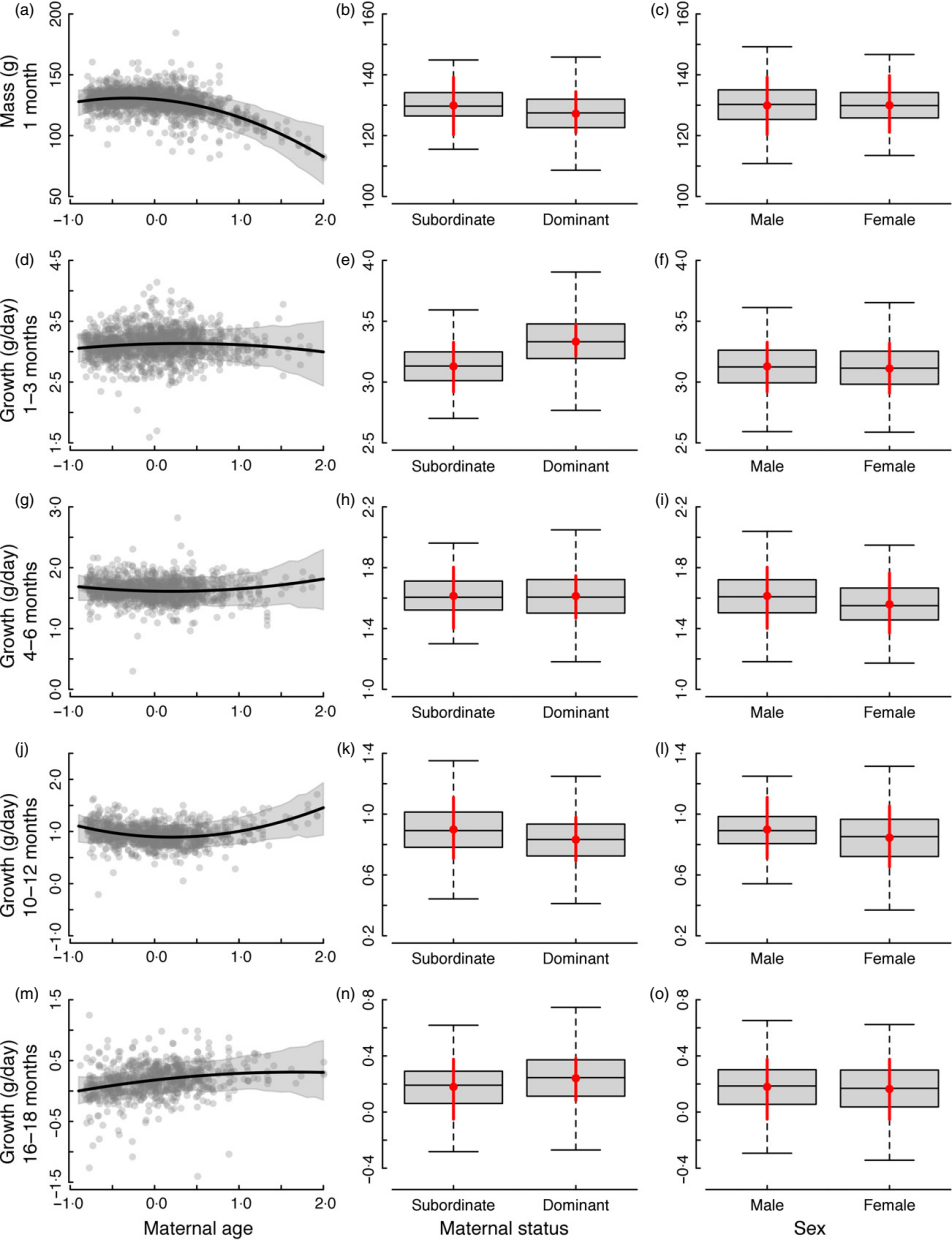
Maternal and individual factors affecting (a–c) mass at 1 month and (d–o) growth at subsequent stages, with left panel displaying the effect of maternal age at conception (standardized), middle panel displaying effect of maternal status and right panel displaying effect of sex. Shown are the predicted mean effects and 95% credible intervals for the model fit to each period (line and shaded area, left panel; red point and lines, middle and right panel). The grey points (left panel) and boxes (middle and right panel) display partial residuals accounting for other terms in the model.

Dominant and subordinate females produced pups of a similar mass, but pups born to dominant mothers grew faster than their subordinate-born counterparts (Tables1 and 2, Fig. 3). Beyond 3 months of age, there was no subsequent effect of maternal dominance status on growth. Older mothers produced lighter pups at emergence, as indicated by the negative quadratic term for maternal age on body mass at 1 month (Fig. 3). Growth did not vary with maternal age for pups or juveniles, but there was a trend for subadults to grow faster when born to older mothers.

### Sex differences in growth

Males and females had similar mass at emergence and growth as pups. Beyond this age, males grew faster as juveniles and subadults, but by the time they reached 16–18 months, sex differences in growth had disappeared (Tables1 and 2, Fig. 3).

### Random-effects variance

The variance explained by random terms across all five analyses suggests that litter-of-origin effects continued to influence growth throughout development, while current group identity generally did not explain any variation in growth and maternal identity was only important for mass at emergence and pup growth (Tables1 and 2). Repeatability estimates for each random-effects level are provided in the Table S1 (Supporting information).

## Discussion

Here, we provide a systematic comparison of the relative influence of abiotic, social and maternal environmental effects on growth across different life stages in wild meerkats. We found that recent rainfall is a consistently important driver of variation in growth at all life stages, even when individuals are dependent on carers for food. As predicted, early development was more strongly affected by maternal and social factors than was development at later stages. Early-life effects also had some delayed consequences, with pups born to older mothers tending to grow faster at maturity.

In spite of changes in factors affecting growth across development, the most consistent pattern we found was a positive effect for rain in the past 2 months on growth at all stages. Meerkats inhabit semi-arid regions, where sporadic pulses of rain are strong drivers of invertebrate population dynamics, which form the majority of meerkats' diet (Cumming & Bernard 1997). Our results confirm previous work showing that long-term rainfall influences lifetime mass patterns (English, Bateman & Clutton-Brock 2012), although we selected a shorter window of rainfall. A shorter window is more appropriate for measuring effects on growth than mass, as rainfall effects on growth over the short term translate into longer-term effects on mass. We also found that seasonal variation influenced growth during the periods of highest growth rate (pups, juveniles and subadults), with growth peaking during the summer and lowest during winter, consistent with findings by English, Bateman & Clutton-Brock (2012).

We predicted that abiotic effects may be weaker when individuals are dependent on carers for food, as buffering effects of carers have been demonstrated in other systems (Cadby, Jones & Wapstra 2011), including cooperative breeders (Covas, du Plessis & Doutrelant 2008). Although rain had a consistent effect on mass and growth at all stages, such buffering may be evident in the lack of seasonal variation in mass at emergence. Mothers might counterbalance direct seasonal effects by adjusting allocation during pregnancy (Sharp, English & Clutton-Brock 2013), for example producing smaller litters during months of low food resources. However, it is also possible that fitting an annual, sinusoidal term does not fully capture the resolution of seasonal variation at the age of emergence. Meerkats are generally seasonal breeders, and lack of data across the year may obscure a seasonal effect on early-life measures of condition.

While abiotic environmental factors had a relatively consistent effect across life stages, social influences on growth had varying patterns. Pups born in larger groups were slightly lighter, suggesting an effect of either food competition among breeding females or adaptive maternal strategies. If mothers produce lighter pups because they are resource-limited, such competition may be an important factor in reproductive suppression, as in banded mongooses (Nichols *et al.* 2012). Alternatively, mothers may strategically produce lighter pups in anticipation of the compensatory effect of having more helpers, as shown in other cooperative breeders (Russell *et al*. 2007a). The production of lighter pups in larger groups is further supported by recent evidence that dominant female meerkats gain less weight during pregnancy in larger groups (Sharp, English & Clutton-Brock 2013). Manipulation experiments are required to establish whether this effect is due to constraints or adaptive strategies by mothers. For example, Dantzer *et al*. (2013) recently manipulated perceived population density while holding resources constant to demonstrate such anticipatory maternal effects in red squirrels.

The period in which social effects were most different to other stages was that of pup growth, consistent with the observation that the best-fitting lifetime growth model incorporates a change in growth rate before and after nutritional independence (English, Bateman & Clutton-Brock 2012). As pups are almost entirely dependent on adults for food, it is not surprising that the number of adults in a group had a positive effect on pup growth, as shown previously by Russell *et al*. (2002). Pups are also in direct competition with their littermates over access to helpers or food (Hodge, Flower & Clutton-Brock 2007) and consequently grew slightly more slowly in larger litters. More unexpectedly, the number of adults in a group had a negligible effect on growth at later stages of development. This lack of effect suggests that larger groups confer neither increased food competition nor benefits of increased vigilance that translate into changes in body mass. Given that meerkats forage independently and the frequency of overt competition over food is low (T. Flower, unpublished data), social factors may be less relevant to growth beyond nutritional independence.

Maternal factors were generally more important in early-life stages, in line with studies showing a decline in maternal effects with age (Lacey & Herr 2000; Lindholm, Hunt & Brooks 2006). Although pups born to dominant mothers were similar in mass to their subordinate-born counterparts, they grew faster during the period of pup dependence. This could be a consequence of the benefits of being born in single-mother litters, which is more likely in dominant breeding attempts (Clutton-Brock *et al.* 2010) or, in the case of mixed-maternity litters, if dominant-born pups emerge earlier and therefore have a size advantage. Pups born to older females were lighter at 1 month of age. This result supports recent evidence for reproductive senescence in meerkats (Sharp & Clutton-Brock 2010), although the trend for pups born to older mothers to exhibit faster growth at 10–12 months suggests that they may compensate for this initial disadvantage. Such compensation for poor maternal quality has, to our knowledge, yet to be demonstrated in a cooperatively breeding system and highlights the importance of considering the processes affecting growth across several stages of development to elucidate complex delayed effects.

A pup's litter of origin explained a considerable proportion of the random-effects variance in growth across development (between 0·5 and 0·8) and maternal identity explained 0·2 of the random-effects variance in mass at 1 month, in contrast to current group identity (less than 0·01 across stages, Table S1, Supporting information). Some of this variation may be accounted for by additive genetic variance, which is currently being investigated elsewhere. Given that body mass growth is highly plastic, however, other aspects of the early maternal, social and abiotic environment not considered in the current analysis may shape development in the long term in this species. Mothers may differ in quality beyond variation in dominance status and age, for example, and given the high variation among individuals in cooperative behaviour (Madden *et al.* 2009; English, Nakagawa & Clutton-Brock 2010), the number of helpers may not entirely encompass the early social environment experienced by young.

The implications of our results – that environmental effects on growth vary across time – for long-term phenotypic development benefit from an appreciation of how energy acquisition and allocation mechanisms themselves vary across development (Hou *et al.* 2008). Earlier on, somatic growth involves structural change, whereas, on reaching asymptotic mass, growth is more reflective of short-term change in condition. As such, environmental effects acting at early stages may have irreversible consequences for later phenotype, whereas those that are important in later life may involve higher levels of flexibility.

In meerkats, there is strong selection on traits associated with dominance acquisition as reproductive skew is high and dominance tenure is long (Griffin *et al.* 2003; Clutton-Brock *et al.* 2006; Sharp & Clutton-Brock 2011). Body mass tends to be more strongly associated with the acquisition of dominance in females than males (Hodge *et al.* 2008; Spong *et al.* 2008), yet here we found that males had faster growth during the subadult period than females. At this stage, males may increase their body condition to start reproduction as early as possible through extra-territorial prospecting forays (Young, Spong & Clutton-Brock 2007), whereas females may be constrained from gaining weight to avoid being evicted by the dominant female (Kutsukake & Clutton-Brock 2005). Previous studies have shown that early body condition influences later survival and reproduction in meerkats (Russell *et al*. 2007b; Hodge *et al.* 2008), yet it is unclear whether such an effect is due to individuals exhibiting faster growth trajectories or reaching a higher body mass at maturity and whether such effects differ between the sexes. These questions are currently being investigated.

In summary, we show here that, while abiotic factors remain a consistent driver of patterns of growth across life stages in wild meerkats, social and maternal effects on growth varied in their influence. The period of nutritional dependence was most sensitive to social factors and direct maternal effects on growth were stronger at younger stages. Comparing changes in the relative influence of abiotic, maternal and social factors across development reveals complex processes affecting growth, such as how carers only provide a positive influence when individuals are nutritionally dependent and that negative maternal effects may be compensated for later in life. Understanding such complexities in the role of environmental factors on trait dynamics may be important for predicting population responses to environmental change (Ozgul *et al.* 2010).

## Supporting Information

Data S1 Data deposited in the Dryad repository: doi:10.5061/dryad.cf033. **Fig. S1.** Linear interpolation of growth (black lines) during the focal windows of interest (shaded in grey). **Fig. S2.** Schematic outlining timescale for growth and environmental measurements. **Table S1.** Repeatability values and 95% credibility intervals for levels of the random effects in each model.
