# Supplementary material for: Maternal, social and abiotic environmental effects on growth vary across life stages in a cooperative mammal
Source: J Anim Ecol. 2013 Nov 18;83(2):332–42. doi: 10.1111/1365-2656.12149 (PMC4286004; doi:10.1111/1365-2656.12149)
Supplement: Data S1 — Data deposited in the Dryad repository: doi:10.5061/dryad.cf033. Fig. S1. Linear interpolation of growth (black lines) during the focal windows of interest (shaded in grey). Fig. S2. Schematic outlining timescale for growth and environmental measurements. Table S1. Repeatability values and 95% credibility intervals for levels of the random effects in each model. [file jane0083-0332-SD1.docx]

**Supplementary Information for "Maternal, social and abiotic environment effects on growth vary across life stages in a cooperative mammal"**

S. English_­,_ A. W. Bateman, R. Mares, A. Ozgul and T. H. Clutton-Brock

*Timescale for growth measurements*

We measured growth as the difference in body mass across two-month windows at specific stages of development, i.e. between 1–3, 4–6, 10–12 and 16–18 months of age. To our estimation, a two-month window allowed for a good balance between resolution for a particular stage and generality across stages. Although the precise nature of growth in the long-term is nonlinear (English, Bateman & Clutton-Brock 2012), a linear approximation performs well over the short term (figure S1).

**Figure S1.** Linear interpolation of growth (black lines) during the focal windows of interest (shaded in grey). Points denote average mass at each age for 776 individuals with growth measurements across all four periods. The red dashed line displays a biphasic, monomolecular growth curve applied to the average mass data.


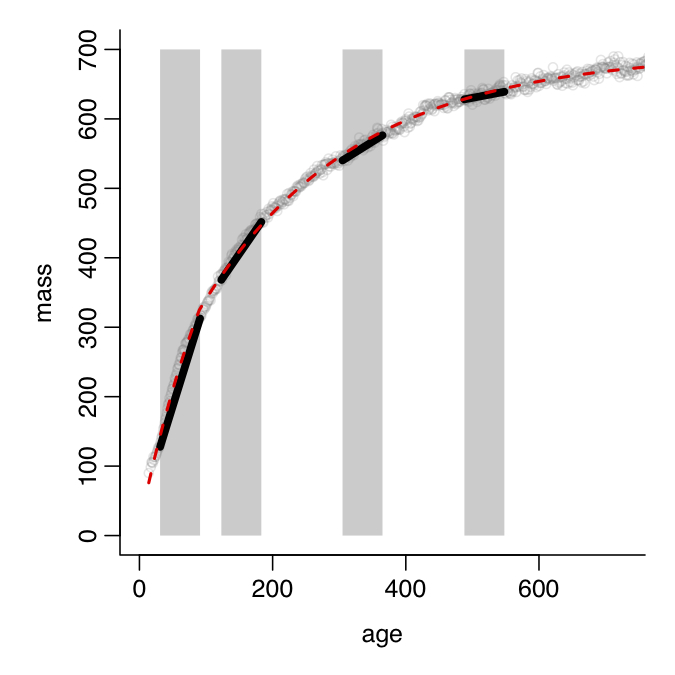


*Timescale for environmental variable measurements*

Most of the environmental variables we considered were measured over a two-month window until the midpoint of the focal period of interest (figure S2). We deemed this window to be appropriate to cover a one-month lag between rainfall and the emergence of invertebrate prey items (Cumming & Bernard 1997). We selected our social measures across the same window for consistency. As group composition is relatively stable at a monthly time-scale, these measures are likely to be unchanged if considered within the focal period of interest as well.

**Figure S2.** Schematic outlining timescale for growth and environmental measurements. For each window, growth is calculated as the difference between mass across a two-month period, i.e.: mass_1_ – mass_0_­, where mass­_1_ is measured at 3, 6, 12 and 18 months respectively. For the analysis of variables affecting mass at emergence (1 month), mass at 1 month of age is equivalent to mass_1_ in this schematic.


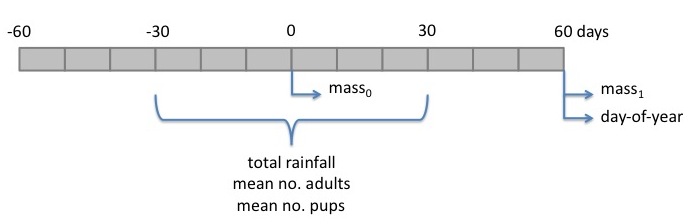


**Table S1.** Repeatability values and 95 per cent credibility intervals for levels of the random effects in each model.

|  | **Mass at 1 m** | **Growth 1-3 m** | **Growth 4-6 m** | **Growth 10-12 m** | **Growth 16-18 m** |
| --- | --- | --- | --- | --- | --- |
| Litter | 0.549  [0.436-0.645] | 0.681  [0.575-0.747] | 0.803  [0.747-0.835] | 0.825  [0.729-0.863] | 0.666  [0.594-0.713] |
| Mother | 0.236  [0.117-0.356] | 0.040  [0.002-0.138] | 0.003  [0.001-0.045] | 0.003  [0-0.034] | 0.003  [0-0.036] |
| Group | 0.0004  [0.000-0.217] | 0.004  [0.001-0.114] | 0.003  [0.001-0.032] | 0.010  [0.001-0.126] | 0.005  [0.001-0.037] |
